# Establishing *Ingrischana* gen. nov. as a First Step in Reviewing Asian Tetriginae (Orthoptera: Tetrigidae) †

**DOI:** 10.3390/life16050797

**Published:** 2026-05-10

**Authors:** Madan Subedi, Josip Skejo

**Affiliations:** 1 Agriculture Science Center, Directorate of Research and Extension, Agriculture and Forestry University, Ghyalchok, Gorkha 34000, Nepal; madansubedi13@gmail.com; 2 Evolution Lab, Division of Zoology, Department of Biology, Faculty of Science, University of Zagreb, Rooseveltov trg 6, 10000 Zagreb, Croatia

**Keywords:** groundhoppers, Inner Terai, pygmy grasshoppers, Tetrigini, *Tetrix*, mitogenomes, phylogeny

## Abstract

A new genus, *Ingrischana* gen. nov. (Tetrigidae: Tetriginae) is established for winged Tetriginae from Asia with extremely setose mid femur, and toothed dorsal margin of the hind femur. Till now, many species of this genus have been erroneously assigned to the genera *Bannatettix* Zheng, 1993; *Formosatettixoides* Zheng, 1994; *Ergatettix* Kirby, 1914; *Euparatettix* Hancock, 1904; *Paratettix* Bolívar, 1887, and *Tetrix* Latreille, 1802. Altogether 2 new species, 11 new combinations, 1 new name, and 2 new synonyms are proposed, and 1 species is reinstated. Two new species are *I. motbotawa* gen. et sp. nov. (Brija Furry Groundhopper) and *I. aspinosa* gen. et sp. nov. (Toothless Furry Groundhopper), both from Nepal. New combinations are *I. aptera* (Zheng et Ou, 2009) comb. nov., *I. barbifemura* (Zheng, 1998) comb. nov., *I. curvimargina* (Zheng et Deng, 2004) comb. nov., *I. dentifemura* (Zheng, Shi et Luo, 2003) comb. nov., *I. grossifemura* (Zheng et Jiang, 1997) comb. nov., *I. longzhouensis* (Zheng et Jiang, 2000) comb. nov., *I. obesa* (Bolívar, 1887) comb. nov., *I. serrifemora* (Deng, Zheng et Wei, 2008) comb. nov., *I. serrifemoralis* (Zheng, 1998) comb. nov., *I. serrifemoroides* (Zheng et Jiang, 2002) comb. nov., and *I. torulosinota* (Zheng, 1998) comb. nov. The new name is *I. parlungana* nom. nov., proposed for *Bannatettix serrifemoralis* Zheng et Shi, 2009a, because of the homonymy with *I. serrifemoralis* (Zheng, 1998) comb. nov. *Ingrischana jhapana* (Ingrisch, 2001a) stat. rev. et comb. nov. is reinstated as a valid species. Two new synonyms are *Formosatettixoides guangxiensis* Zheng & Jiang, 2000 syn. nov. (of *I. longzhouensis* comb. nov.), and *Ergatettix serifemoroides* Zheng et Shi, 2009b syn. nov. (of *I. parlungana* nom. nov.). The new genus is defined not only by morphological apomorphies, but is also confirmed by mitogenome phylogeny.

## 1. Introduction

The groundhopper subfamily Tetriginae is the largest subfamily within the Tetrigidae (Orthoptera: Caelifera) family, with a fascinating 655 species. Interestingly, even 75%, and that is 491 species, belong to one of the eight taxonomically complex genera: *Tetrix* Latreille, 1802 [1] (136 spp.), *Euparatettix* Hancock, 1904 [2] (71 spp.), *Coptotettix* Bolívar, 1887 [3] (71 spp.), *Formosatettix* Tinkham, 1937 [4] (70 spp.), *Paratettix* Bolívar, 1887 [3] (53 spp.)*, Hedotettix* Bolívar, 1887 [3] (50 spp.), *Ergatettix* Kirby, 1914 [5] (19 spp.), and *Alulatettix* Liang, 1993 [6] (18 spp.) ([7], this study).

During our recent research on Nepali Tetrigidae, we have already faced an issue of a new genus that was hidden under *Coptotettix* [8], and again, we encountered a similar issue. Namely, at the moment of the discovery of two new species we report in this study, we were unable to definitely assign them to any of the aforementioned Tetriginae genera. There are *Bannatettix* Zheng, 1993 [9]; *Formosatettixoides* Zheng, 1994 [10]; *Ergatettix*; *Euparatettix*; *Paratettix,* and *Tetrix* species [3,11,12,13,14,15,16,17,18,19,20,21] with characters similar to the ones found in our new species—extremely setose mid femora, and dentate dorsal margin of the hind femora. As these characters are not usually exhibited among members of Tetriginae, we realized we are dealing with a new, undescribed genus.

The current study is based on an independent faunistic survey conducted in Nepal between March 2024 and September 2025, employing opportunistic sampling across a broad elevational gradient (120–755 m a.s.l.) spanning the Terai lowlands and lower Himalayan foothills. These regions are characterized by a humid subtropical climate under strong south-eastern monsoon influence [22]. Although Tetriginae are characteristic components of these ecosystems, their diversity in Nepal remains insufficiently documented. Existing records are sparse and largely restricted to the vicinity of Satighat, where a few species have been reported (*Coptotettix conspersus* Hancock, 1915 [23], *Hedotettix gracilis* (de Haan, 1842) [24], and *Paratettix variabilis* Bolívar, 1887 [3]) [25], while other surveyed localities (Lake Khaste, Lake Brija, and the Tamagadhi forest–agricultural mosaic) have remained entirely unexplored.

Groundhoppers play key ecological roles as primary consumers and detritivores, contributing to nutrient cycling and supporting trophic networks [26]. The absence of targeted surveys in these habitats thus represents a significant gap in our understanding of Himalayan biodiversity.

The aim of this paper is to define the genus *Ingrischana* **gen. nov**., by the number of species—that being 15—currently the ninth largest within Tetriginae; to find its placement in the Tetrigidae tree of life using mitogenome phylogeny; to describe two new species of this interesting Tetriginae genus from Nepal; and to briefly review species hitherto assigned to *Tetrix*, *Paratettix*, and *Ergatettix*, but which should be moved to *Ingrischana*.

## 2. Materials and Methods

We have consulted the original descriptions [3,11,12,13,14,15,16,17,18,19,20,21] of all the species we deal with in this study, as well as all available type specimens or their photographs. Taxonomy follows Orthoptera Species File [7], and the nomenclature is in accordance with the International Code of Zoological Nomenclature [27]. The holotype of *Ingrischana motbotawa* gen. et sp. nov. was opportunistically collected by the first author near Lake Brija (27.67129° N, 82.96422° E) during an incidental survey in March 2024. Similarly, the paratype was collected during an opportunistic field visit to Satighat, Tumlingtar (27.31171° N, 87.19885° E) in August 2025. Specimens of new species reported in this study were pinned using Phusis stainless steel insect pins (size #0) and deposited at the Annapurna Natural History Museum, Pokhara, Nepal and the Insect Collection of Agriculture Science Center, Ghyalchok, Gorkha, Nepal. The images of the individuals were taken post-collection by a Canon EOS 80D camera (Canon Inc., Tokyo, Japan) with a Canon EF 100 mm f/2.8 USM macro lens (Canon Inc., Tokyo, Japan), and were post-processed with the software Adobe Photoshop CS6 version 13.0.1 [28]. Videos of habitats of the species (Lake Brija and Satighat) were taken with a Nothing 1 phone camera (Nothing Technology Limited, London, UK), and the video from Tamagadhi was taken with a Vivo V27e phone camera (Vivo Mobile Communication Co., Ltd., Dongguan, China). The videos were uploaded to the YouTube channel ‘Nepali Grasshoppers’ (https://www.youtube.com/@nepaligrasshoppers (accessed on 8 October 2025)). The video links are provided in the appropriate parts of the text. Morphological terminologies follow [29,30]. The measurements of the vertex and eye follow [31] while other measurements follow [30,32]. Measurements were made with the software ImageJ v1.53k [33] by calibrating the images with millimeter paper. ChatGPT version GPT-5.1 (accessed on 12 March 2026), an AI-based language model developed by OpenAI (San Francisco, CA, USA), and DeepSeek-V3 (accessed on 12 March 2026), an AI-based model developed by Hangzhou DeepSeek Artificial Intelligence Basic Technology Research Co., Ltd. (Hangzhou, China), were used for grammar and syntax corrections during the preparation of this manuscript.

Sequences of whole or partial mitogenomes of 30 Tetrigidae species were downloaded from the National Center for Biotechnology Information (NCBI), based on previously published studies [34,35,36,37,38,39,40,41,42,43,44,45]. The dataset included one Batrachideinae species (outgroup), 7 Criotettiginae species (6 genera), 4 Scelimeninae species (2 genera), one Xerophyllini member, and 11 Tetriginae species (10 genera), of which 7 species belong to Tetrigini. One member of *Ingrischana* gen. nov. has been included in the analysis in order to test its position, that being *I. serrifemora* comb. nov. (Table 1).

Mitogenome sequences were aligned using the MAFFT multiple sequence alignment program (version 7.0; [46]) via the online server (https://mafft.cbrc.jp/alignment/server/index.html (accessed on 11 April 2026)). The final alignment comprised 21,321 sites; 9034 sites were found to be parsimony informative; 3772 were singleton sites; and 8735 were constant sites. Pairwise genetic distances were calculated in MEGA version 12 [47] using the Maximum Composite Likelihood method, accounting for both transitions and transversions.

A maximum likelihood phylogram was reconstructed using IQ-TREE version 3.0.1 [48,49] under the Generalized Time Reversible model TREE [50] with a gamma distribution of rate heterogeneity and a proportion of invariant sites (GTR + I + G). Node support was calculated using 1000 ultrafast bootstrap replicates (UFBoot) and 1000 SH-like approximate likelihood ratio test (SH-aLRT) replicates, with the -bnni option applied to improve node support accuracy. The resulting tree was rooted on *Saussurella* sp. (Batrachideinae). The tree was visualized in the Interactive Tree of Life (iTOL) [51,52].

## 3. Results

### 3.1. Taxonomy

**Figure 1:**
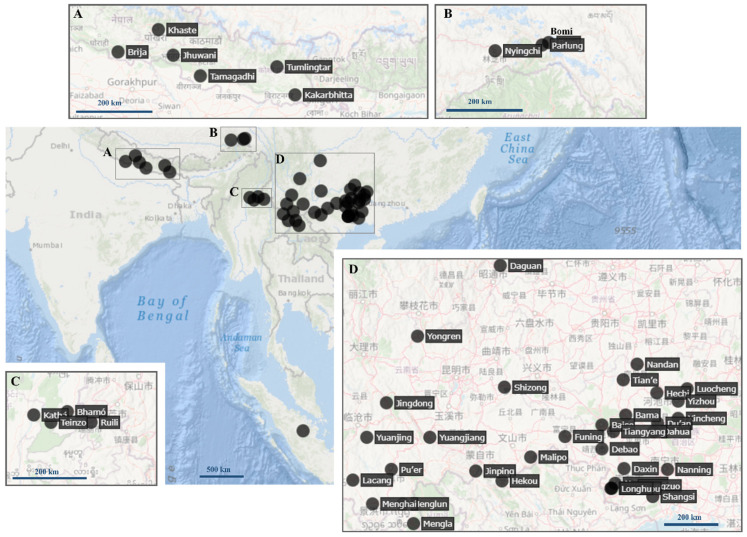
Distribution of the genus *Ingrischana* **gen. nov**. (**A**) Himalayan region; (**B**) Southeastern Tibetan Plateau; (**C**) Northern Myanmar and bordering Yunnan; (**D**) South China. All localities mentioned in this study are shown; a doubtful record from Tanzania is not included in the map. Locality in Sumatra is not specified, while a record from Tanzania has been omitted. The map was generated using GPS Visualizer [56].
  **Diagnosis.** Head, pronotum and legs morphology typical for Tetriginae genera, but specific in: (i) vertex subequal to a compound eye or wider, apex truncated in dorsal view; (ii) tip of the fastigium not projected before the compound eyes; (iii) mid femora widened; (iv) fore and mid femora covered in numerous hairs; (v) serrated dorsal and/or margins of the hind femora; (vi) most commonly in a brachypronotal state (except for *I. serrifemoroides* comb. nov. and *I. serrifemoralis* comb. nov. which are pauropronotal; and *I. obesa* comb. nov. may be brachypronotal and macropronotal); and (vii) toothed pulvilli of the hind tarsi.
  **Comparison with *Paratettix***. The new genus differs from *Paratettix* Bolívar, 1887 [3] (based on the type species, *Paratettix meridionalis* (Rambur, 1838) [51]), to which the type species of *Ingrischana* gen. nov. was previously assigned [3,57] by the following characters: (i) continuous median carina (median carina broken/missing in prozona in *Paratettix*); (ii) strongly setose mid femora (hairless or weakly setose in *Paratettix*), and (iii) ventral and/or dorsal margins of the hind femora with toothed serrations (margins of hind femora smooth in *Paratettix*).
  **Description**.
  **Head:** *In dorsal view* vertex subequal to an eye width or wider and finely granulated; anterior margin of the vertex truncated, i.e., in level with or not reaching the anterior margin of the eyes; transverse and lateral carinae of the vertex forming rounded right angle, i.e., L-shape with rounded angle; medial carina distinctly elevated. In frontal view frontal costa short, bifurcating in the upper third of the eye height; facial carinae typically widened downwards; face covered with fine hairs. In lateral view head not exserted above the pronotal surface, but at the level of it or slightly below; vertex visible above the eyes; occipital area narrow; angle between frons and vertex obtuse rounded; facial carinae slightly convex, protruding in front of the anterior level of eyes.
  **Antenna**: Filiform, composed of 14–15 antennomeres
  **Pronotum:** In dorsal view, surface rugose, covered in small nodules, wrinkles, or tubercles; prozonal carina subparallel to converging posteriorly; lateral lobes lack spines and are directed downwards; shoulders broad, humeroapical carina forming with lateral carina wide rounded angle. In the frontal view characteristically tectiform (roof-like) with an elevated median carina. In lateral view, predominantly brachypronotal (not exceeding hind knees), rarely pauropronotal, and macropronotal; infrascapular area wide, trapezoidal, usually reaching half of the hind femur length.
  **Wings:** Tegmina and alae generally present; most species brachypronotal; some macropronotal or pauropronotal; some species have several states.
  **Legs:** Robust. Densely covered in hairs. Mid femura swollen, covered in dense hairs. Hind femora dorsal margin always serrated (saw-like); ventral margin usually less serrated than dorsal with undulating margins; first segment of hind tarsi longer than the third; pulvilli of the hind tarsus triangular and acute with apical teeth.
**Catalog of the species belonging to *Ingrischana* gen. nov.**
***Ingrischana aptera* (Zheng et Ou, 2009) comb. nov.**
  =*Euparatettix apterus* Zheng et Ou, 2009 [19]
  **Distribution**: China: known from two localities in Yunnan (Ruili, Yingjiang) [19,58].
  **Notes**. Transferred from the genus *Euparatettix* Hancock, 1904 [2].
***Ingrischana aspinosa* Subedi et Skejo gen. et sp. nov.**
  **LSID.** urn:lsid:zoobank.org:act:DB77DBFE-992A-4001-A88B-D0436733C9AA
  **Vernacular name:** Toothless Furry Groundhopper, based on the blunt ovipositor teeth in females, and based on the generic vernacular name (Asian Furry Groundhoppers).
  **Etymology:** The specific epithet *aspinosa*, meaning ‘lacking spines’, is derived from Latin, *a-* meaning ‘without’, and ‘*spinosus*’ meaning ‘with spines’. The name refers to the blunt or reduced ovipositor teeth, distinguishing this species from its congeners with more pronounced teeth (spines).
  **Type material: Holotype** 1♀, NEPAL: Madhesh Province: Bara district: Kolhabi Municipality: Tamagadhi: Herbs Production & Processing Co. Ltd.: Agricultural fields; 27.09552° N, 85.15825° E; ca. 120 m a.s.l.; 16.vii.2025; M. Subedi leg.; collected by an aerial net; ICAG (ICAG-ORT-TETR214).
  **Distribution.** Known only from the type locality (Figure 1 and Figure 2G,H).
  **Habitat description.** The habitat is agricultural land primarily used for the commercial production of aromatic and medicinal herbs (Figure 2D,H). The video of the type locality can be viewed at https://www.youtube.com/shorts/ol8wFP6YPKA?feature=share (accessed on 30 July 2025).
  **Diagnosis.** The main difference between *I. aspinosa* gen. et sp. nov. and other species of *Ingrischana* gen. nov. is in the morphology of the ovipositor. This species has blunt ovipositor teeth, while others have sharp ovipositor teeth. Comparison with other *Ingrischana* species is shown in Table 3 and Table 4.

Family Tetrigidae Rambur, 1838 [53]
  Subfamily Tetriginae Rambur, 1838 [53]
  **Justification of the placement.** Members of *Ingrischana* gen. nov. exhibit typical Tetriginae characteristics: (i) L-shaped carina of the vertex; (ii) lateral lobes directed downwards and contiguous with the body; (iii) dorsal margin of anterior and mid femora carinate (not sulcate); (iv) median ocellus below the lower margin of the eyes; and (v) presence of a filiform antenna.
**Genus *Ingrischana* gen. nov.**
  **LSID.** urn:lsid:zoobank.org:act:5337DA82-2DE8-4215-A540-E44FE8A5596E
  **Derivatio nominis.** Patronymic. The genus is named in honor of Dr. Sigfrid Ingrisch, a renowned German orthopterist, a pioneer of Nepali Orthopterology, and our mentor. Ingrisch has described more than 750 Orthoptera taxa worldwide and his papers (e.g., [14,25,54,55] inspired many young scientists to study grasshoppers and crickets. The name is formed as a feminine noun in apposition.
  **Vernacular name:** Asian Furry Groundhoppers, based on the occurrence (Asia), and based on the hairy appearance of the mid and hind femora.
  **Type species:** *Paratettix obesus* Bolívar, 1887 [3] (=*Ingrischana obesa* comb. nov.) by present designation. Holotype female, most likely from Myanmar, deposited at the NMW.
  **Composition and distribution:** Currently includes 15 species (Table 2, Figure 1.) inhabiting Nepal, China (Guangxi, Yunnan, Tibet), Myanmar, and Thailand ([7], this study).

**Figure 2:**
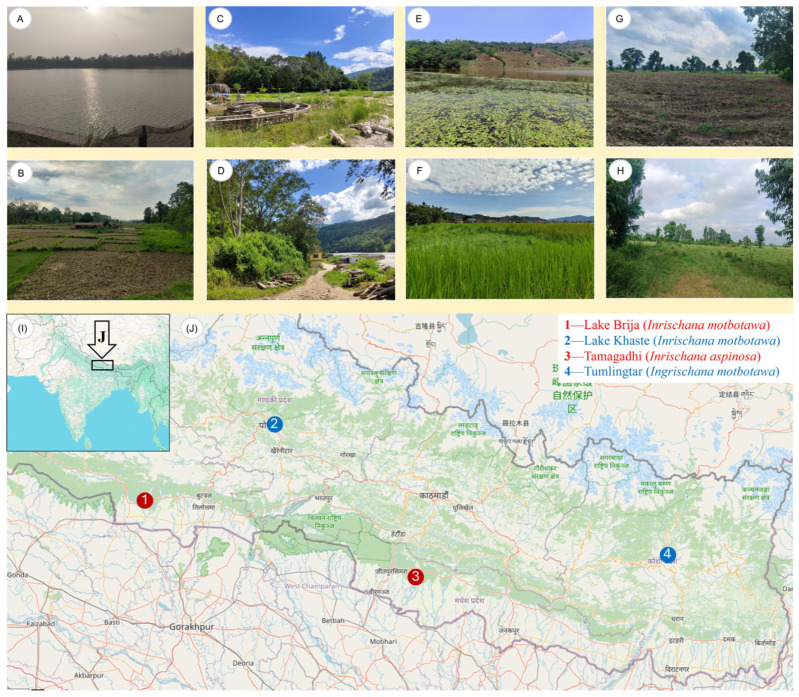
Habitat (**A**–**G**) and distribution (**I**,**J**) of *I. motbotawa* gen. et sp. nov. (**A**–**F**) and *I. aspinosa* gen. et sp. nov. (**G**,**H**) from Nepal. (**A**) Lake Brija, the type locality of the former species, and (**B**) adjacent agricultural fields; (**C**) plains on the bank of the Arun River and (**D**) the periphery of Satighat, Tumlingtar, Khandbari, Sankhuwasabha; (**E**) lake Khaste, Pokhara, Kaski, and (**F**) adjacent agricultural fields. (**G**) Agricultural fields in Tamagadhi, Bara, type locality of the latter species, prepared for the plantation of medicinal and aromatic herbs and (**H**) the fields with ongoing plantation. The map shows the position of type localities in red, and paratype localities in blue. Broader (**I**) and narrower (**J**) contexts are shown. The map was generated using GPS Visualizer [56].
  **Description.** Female Holotype (Figure 3 and Figure 4)
  **Head.** In the dorsal view. Surface of the vertex finely granulated. Vertex between the eyes 1.25× wider than a compound eye. Vertex at the base of the eyes 1.77× wider than an eye; narrowing anteriorly and subequal to the width of an eye at its apex. Anterior margin of the fastigium truncated, not reaching the anterior margin of the compound eyes. Medial carina not protruding in front of the compound eyes, distinctly elevated and extending nearly to the base of head. Transverse and lateral carinae of the vertex forming rounded right angle (L-shape with rounded angle). Fossula shallow, elongate, reaching the posterior margin of the medial carina. Eyes reniform. In the frontal view. Face covered with sparse fine hairs. Top margin of eyes slightly above the vertex. Frontal costa short, bifurcating in the upper third of a compound eye height. Facial carinae progressively widen downwards, then slightly narrowing below superior ocelli, and then progressively widen again toward the median ocellus. Scutellum between the antennal grooves slightly narrower than an antennal groove. Paired ocelli located at mid-height of the eyes, slightly below the point of the frontal costa bifurcation. Top margin of antennal groove above the lower margin of eyes; bottom margin slightly below the lower margin of eyes. In the lateral view. Head placed below the level of pronotal surface (below the highest point of median carina). Angle between frons and vertex obtuse rounded. Facial carinae slightly convex, protruding in front of the anterior level of eyes.
  **Antenna.** Filiform. Composed of 15 antennomeres (scape, pedicel and 13 flagellar antennomeres). Mid antennomeres around 7.5× as long as wide. One antenna as long as the distance between the anterior margin of the vertex and the mid-length of tegmina.
  **Pronotum.** Slender and brachypronotal species with finely granulated/wrinkled pronotum, tip of the pronotum not exceeding hind knees. In the dorsal view. Anterior margin of pronotum produced between prozonal carinae. Median carina continuous, reaching the apex of the pronotum. Surface rough; covered with small nodules and wrinkles. Prozonal carinae elevated, subparallel. Humero-apical carinae distinctly visible, forming with external lateral carina weakly projected rounded humeral angles (shoulders). Interhumeral carina indistinct. Pronotum widest at humeral angles, progressively converging caudad. Lateral lobes bluntly rounded, directed downwards, and contiguous to the body. Spines on the lateral lobes absent. Lateral area wide. Apex of pronotum blunt. In the lateral view. Median carina distinctly elevated in the region of prozona, slightly undulated in the region of metazona, elevated in the posterior third of metazona and then progressively lowering towards the apex. Prozona nodulated. Prozonal carina visible, short. Extralateral carina indistinct. Lateral area wide, narrowing caudally. Apex of lateral lobe subrounded. Infrascapular area subrectangular, widest at the middle. Ventral and tegminal sinus evident, right angled. In the frontal view. Pronotum tectiform (roof-like), with visibly elevated median carina. Lateral lobes of paranota directed downwards and slightly sideways.
  **Wings.** Wings present. Tegmina (forewings) elongated, oval, entirely visible. Alae (hindwings) not reaching the apex of the pronotum.
  **Legs.** All legs covered with sparse hairs and with distinct black rings. *Fore legs:* Femora robust. Dorsal margin of femur convex; posterior fourth of ventral margin slightly concave, rest straight. Tibia with few acute spines on the ventral distal surface. *Mid legs:* Femora robust, widest at the middle. Dorsal margin of femur slightly convex, except concave towards the distal end. Tibiae with acute spines on the ventral margin. *Hind legs:* Femora robust. Dorsal and median external areas with a series of parallel transversal ridges. Dorsal margin undulating with a few minute, acute protrusions in the distal half; ventral margin undulating with several minute acute and few blunt teeth. Antegenicular teeth large, triangular and protruded with blunt tip. Genicular teeth large, subtriangular. Tibiae smooth with several blunt spines. First tarsal segment longer than third. Pulvilli triangular and acute; distal pulvillus longest.
  **Ovipositor.** Covered with sparse hairs. Ovipositor valves widened, apices blunt. Dorsal valves with blunt serrations; ventral valves lack serrations (Figure 5A).
  **Sexual dimorphism.** Unknown. Only female of *I. aspinosa* sp. nov. is currently known; therefore, sexual dimorphism cannot be assessed and remains to be documented when additional material becomes available.
  **Measurements:** See Table 5.
***Ingrischana barbifemura* (Zheng, 1998) comb. nov.**
  =*Tetrix barbifemura* Zheng, 1998 [12]
  =*Tetrix barbifemora* [sic] Zheng, 1998 in [59], misspelling of *T. barbifemura*
  **Distribution**: China: reported from multiple localities in Yunnan (Mengla (Figure 6), Menghai, Menglun, Pu’er, Yuanjing, Jinping, Lacang, Jingdong) and Guangxi (Baise) [55].
  **Notes**. Transferred from the genus *Tetrix* Latreillle, 1802 [1].
***Ingrischana curvimargina* (Zheng et Deng, 2004) comb. nov.**
  =*Tetrix curvimarginus* Zheng et Deng, 2004 [17]
  **Distribution**: Known only from the type locality [17], (see Table 2.).
  **Notes**. Known only from the holotype female. Transferred from the genus *Tetrix* Latreille, 1802 [1].
***Ingrischana dentifemura* (Zheng, Shi et Luo, 2003) comb. nov.**
  =*Tetrix dentifemura* Zheng, Shi et Luo, 2003 [16]
  =*Tetrix grossus* Zheng, Shi et Luo, 2003 (HT ♀ China: Guangxi: Xincheng (SNNU)), synonymized with *T. dentifemura* by [60]
  =*Tetrix grossus* Zheng et Shi, 2003, wrong authorship cited by [61]
  =*Tetrix dentifemorus* [sic] Zheng, Shi et Luo, 2003 in [61], misspelling of *T. dentifemura*
  *=Tetrix dentifemora* [sic] Zheng, Shi et Luo, 2003 in [58], misspelling of *T. dentifemura*
  **Distribution**: China: reported from many localities in Yunnan (Malipo, Hekou, Funing, Shizong, Daguan), and Guangxi (Dahua, Du’an, Bama, Daxin, Debao, Baise, Xincheng, Tian’e, Yizhou, Hechi, Nandan) [61]. Previously reported only from Guangxi (Xincheng) as pauropronotal *I. dentifemura*, and from all other localities as its synonym, brachypronotal *Tetrix grossus* (e.g., [61]).
  **Notes**. Transferred from the genus *Tetrix* Latreille, 1802 [1].
***Ingrischana grossifemura* (Zheng et Jiang, 1997) comb. nov.**
  =*Tetrix grossifemura* Zheng et Jiang, 1997 [11]
  =*Tetrix gossifemura* [sic] Zheng et Jiang, 1997 in [61], misspelling of *T. grossifemura*
  **Distribution**: China: known from two localities in Guangxi (Nanning, Longzhou) [11,61].
  **Notes**. Transferred from the genus *Tetrix* Latreille, 1802 [1].
***Ingrischana jhapana* (Ingrisch, 2001a) stat. rev.**
  **Distribution**: Known from a single locality in Nepal, Jhapa: Kakarbhitta [14].
  **Notes**: Ingrisch [14] described *Paratettix jhapanus* Ingrisch, 2001a from Kakarbhitta (misspelled as Karkabita in the original text), Jhapa, Nepal. Tumbrinck [62] synonymized this species with *Paratettix obesus* as a brachypronotal variant. We examined the holotype of *P. jhapanus* (SMF-SA 103), deposited in SMF, and found that *P. jhapanus* differs remarkably from *I. obesa* comb. nov. by the following characters: (i) robust body shape; (ii) prozonal carinae parallel or faintly diverging caudad (prozonal carinae converging caudad in *I. obesa* comb. nov.); (iii) anterior margin of vertex in level with anterior margin of eyes (anterior margin of vertex does not reach the anterior margin of eyes in *I obesa* comb. nov.); (iv) In lateral view, the pronotum of *P. jhapanus* has distinctly steeper anterior slope in the prozona descending more abruptly towards the head, while posteriorly, the pronotal apex is upturned (in *I. obesa* comb. nov., the pronotum has gradually sloping prozona and a gently tapering, non-elevated apex, which follows the general outline of the pronotal disk). Based on these distinct characters, *Paratettix jhapanus* is herein revived as a valid species and transferred to the genus *Ingrischana* gen. nov., as its morphological features comply with the new genus.
***Ingrischana longzhouensis* (Zheng et Jiang, 2000) comb. nov.**
  =*Tetrix longzhouensis* Zheng et Jiang, 2000 [13]
  =*Formosatettixoides guangxiensis* Zheng et Jiang, 2000 **syn. nov.** (HT ♀ from China: Guangxi: Longzhou, Longhy; deposited in SNNU) [13]
  **Distribution**: Known only from the type locality in China and its vicinity. Guangxi (Longzhou: Nonggang, Longhu) [61].
  **Notes**. Transferred from the genus *Tetrix* Latreille, 1802 [1]. *Formosatettixoides guangxiensis* Zheng & Jiang, 2000 [13] is hereby synonymized with *I. longzhouensis* (Zheng & Jiang, 2000) comb. nov. Namely, *F. guangxiensis* **syn. nov.** clearly represents a nymph as it lacks an antegenicular notch; and is clearly a nymph of *Ingrischana* gen. nov. based on the hairy fore and mid femoa and serrated margins of the hind femora. Furthermore, this species was caught in the same day on the same locality (Guangxi: Longzhou: Longhu) as *Ingrischana longzhouensis* comb. nov. and published in the same publication (Zheng and Jiang, 2000). Following the Principle of the first revisor ([27]: ICZN Article 24.2), priority is assigned to the name *longzhouensis*. because (1) the type series of *I. longzhouensis* comb. nov. consists of adult specimens, and (2) the description of *T. longzhouensis* (page 144) precedes that of *F. guangxiensis* (page 145).
***Ingrischana motbotawa* Subedi et Skejo gen. et sp. nov.**
  **LSID.** urn:lsid:zoobank.org:act:FAF764F6-BB0E-4FD8-8759-9ED8ED80D8B7
  **Derivatio nominis.** The species epithet *motbotawa* is derived from the local Tharu language, where *mot* means ‘fat’, and *botawa* means ‘grasshopper.’ The name thus translates to ‘*fat grasshopper*,’ referring to the species’ relatively robust body form. It is treated here as a feminine noun in apposition.
  **Vernacular name:** Brija Furry Groundhopper, based on the species’ type locality (Lake Brija) and based on the generic vernacular name (Asian Furry Groundhoppers).
  **Type material. Holotype.** (Figure 7 and Figure 8A–D) 1♀, NEPAL: Lumbini Province: Kapilvastu district: Buddhabhumi Municipality: Brija Lake: Agricultural fields near a lake; 27.67129° N, 82.96422° E; ca. 130 m a.s.l.; 24.iii.2024; M. Subedi leg.; collected by hand; ICAG (ICAG-ORT-TETR211). **Paratype.** (Figure 8E–I) 1♂, NEPAL: Koshi Province: Sankhuwasabha district: Khandbari Municipality: Tumlingtar, Satighat: Banks of Arun river; 27.31171° N, 87.19885° E; ca. 400 m a.s.l.; 22viii.2025; M. Subedi leg.; collected by hand; ICAG (ICAG-ORT-TETR215)|(Figure 8J–L) 1♀, NEPAL: Gandaki Province: Kaski district: Pokhara Metropolitan City: Khaste Lake: Agricultural fields near a lake; 28.19459° N, 84.05241° E; ca. 755 m a.s.l.; 01.ix.2025; M. Subedi leg.; collected by an aerial net; ANHM.
  **Distribution.** Known only from Nepal: Brija Lake (the type locality) in the southern part of the country, the banks of the Arun River in the eastern part, and Khaste Lake in the central part. Khaste Lake lies 120 km northeast of Brija Lake, and Satighat is located 420 km northeast of Brija Lake.
  **Habitat description.** The type locality is the agricultural land adjacent to a lake (Figure 2A,B). The video of the type locality can be viewed at https://www.youtube.com/watch?v=XJgBxG7bnCc (accessed on 24 July 2025). The male paratype’s habitat locality is the riverbank of the Arun River (Figure 2C,D). A video of the locality can be viewed at https://www.youtube.com/shorts/iwucv92x4_A (accessed on 3 September 2025). The female paratype’s habitat locality is an agricultural land adjacent to a lake (Figure 2E,F). The video of the locality can be viewed at https://www.youtube.com/shorts/Cd5pHUEt4BI (accessed on 8 October 2025).
  **Diagnosis.** *Ingrischana motbotawa* gen. et sp. nov. is a winged brachypronotal species with a wide vertex, most similar to *I. obesa* comb. nov. Comparison with all *Ingrischana* species is given in Table 3 and Table 4.
**Description.**
  **Head.** In the dorsal view. Vertex surface finely granulated and covered in hairs. Vertex between the eyes 1.4× wider than a compound eye. Anterior margin of the fastigium truncated, not projected before the compound eyes (anterior margin of vertex in level with anterior margin of eye). Medial carina protruded in front of the compound eyes. Medial carina evident along the apical half of the vertex. Transverse and lateral carinae of vertex forming L-shape with rounded angle. Fossula evident, short and deep, placed in the anterior third of vertex between the eyes. Eyes reniform (oval but more rounded than in most other *Tetrix* species). Occipital area narrow, not wider than a third of a compound eye. In the frontal view. Face covered with fine hairs. Top margin of eyes slightly above vertex. Frontal costa short, bifurcating in the upper third of a compound eye height. Facial carinae progressively widen downwards. Scutellum between the antennal grooves as wide as an antennal groove. Paired ocelli placed halfway of the eye height, slightly below the bifurcation. Top margin of an antennal groove above the bottom margin of eyes, while bottom margin of an antennal groove slightly below the bottom margin of eyes. In the lateral view. Head positioned slightly below the highest point of the pronotal median carina. Angle between frons and vertex obtuse-rounded. Facial carinae slightly convex, protruding anterior to the eyes.
  **Antenna.** Filiform. Composed of 15 antennomeres (scape, pedicel and 13 flagellar antennomeres). Mid antennomeres approximately 5× as long as wide. Each antenna approximately as long as the distance from the anterior margin of vertex to the coxa of mid femur.
  **Pronotum.** Robust and brachypronotal species with finely granulated/wrinkled pronotum, tip of the pronotum not exceeding hind knees. In the dorsal view. Anterior margin of pronotum slightly produced between prozonal carinae. Median carina continuous, reaching the apex of the pronotum. Surface rough; covered with small nodules and wrinkles. Prozonal carinae parallel to slightly converging caudad, distinctly elevated. Humero-apical carinae distinctly visible, forming with external lateral carina weakly projected rounded humeral angles (shoulders). Interhumeral carina indistinct. Pronotum widest at humeral angles, progressively converging caudad. Lateral lobes bluntly rounded, directed downwards, and contiguous to the body. Spines on the lateral lobes absent. Lateral area wide. Apex of pronotum blunt. In the lateral view. Median carina distinctly elevated in the region of prozona, flat in the anterior region of metazona progressively lowering in the posterior third. Prozona wrinkled, moderately nodulated. Prozonal carina clearly visible, short. Extralateral carina indistinct. Lateral area wide, widening caudally. Apex of the lateral lobe subrounded. Infrascapular area subrectangular, widest at the middle. Ventral and tegminal sinus evident, right angled. In the frontal view. Pronotum tectiform (roof-like), with visibly elevated median carina. Lateral lobes of paranota directed downwards and slightly sidewards.
  **Wings.** Wings present. Tegmina (forewings) elongated, oval, entirely visible. Alae (hindwings) do not reach the apex of the pronotum.
  **Legs.** All legs covered with dense hairs and with distinct black rings. *Fore legs:* Femora robust. Dorsal margin of femur slightly convex, with even undulation throughout, except at about one-fourth from the distal end where it forms a distinct raised prominence; posterior fourth of ventral margin slightly concave, rest straight. Tibiae with numerous acute spines on the ventral distal surface. *Mid legs:* Femora robust, widest at the middle. Tibiae with acute spines on the ventral margin. *Hind legs:* Femora robust. Dorsal and median external areas with a series of parallel transversal ridges. Dorsal margin undulating, with numerous minute acute protrusions distally; ventral margin undulating with several distinct teeth. Antegenicular teeth large, triangular with blunt tip. Genicular teeth large, subtriangular. Tibiae smooth with several acute spines. First tarsal segment longer than third. Pulvilli triangular, sharp, with apical teeth; distal one larger than the proximal two in size; the proximal pulvillus shorter than the two distal pulvilli, which are similar in length.
  **Ovipositor.** Covered with dense hairs. Ovipositor valves widened with fine serrations; apices hooked and acute (Figure 5B).
  **Sexual dimorphism.** The female is more robust and slightly larger than the male; however, several body parts are comparatively longer in the male (see Table 3 and Table 4 for detailed morphometric comparisons). The ventral margins of the hind femora bear distinct teeth in the female, whereas these are reduced to fine blunt serrations in the male. The mid femora are more robust in the male, while the infrascapular and lateral areas are relatively wider in the female.
  **Morphological variation.** In the holotype and male paratype, tegmina are elongate and oval in shape, whereas in the female paratype, tegmina are elongate and subtriangular in outline (Figure 9).
  **Measurements:** See Table 5.
***Ingrischana obesa* (Bolívar, 1887) comb. nov. (Figure 10)**
  =*Paratettix obesus* Bolívar, 1887
  =*Paratettix hirsutus* Brunner von Wattenwyl, 1893 [57] (Many syntypes from Myanmar (Sagaing: Kathá; Kachin: Bhamó.; Teinzo) in MHNG, NMW, MHNG), synonymized by Günther [63].
  **Distribution**: Myanmar, Sumatra, and maybe Nepal (Jhuwani, see below). The type locality of *P. hirsutus*—a synonym of *I. obesa* comb. nov.—is Myanmar, whereas for *I. obesa* comb. nov. no locality was specified in the original description [3]. We question the presence of *I. obesa* comb. nov. in Tanzania, which is far away from the rest of the species’ distribution area.
  **Notes**. Transferred from the genus *Paratettix* Bolívar, 1887 [3]. Günther [63] examined the type specimens of both *I. obesus* comb. nov. and *Paratettix hirsutus* and synonymized the two taxa. Ingrisch [55] noted that specimens identified as *P. hirsutus* from Jhuwani (misspelled as “Jhawani” in [55], Nepal, were smaller than the measurements given in [57,64]. We doubt that these specimens truly belong to *I. obesa* comb. nov., as they differ from the holotype of *I. obesa* comb. nov. in having anterior margin of the vertex level with the anterior margin of eyes (in *I. obesa* comb. nov. it does not reach that level), and by the shape of eyes (globular in *I. obesa* comb. nov., oval in the Jhuwani specimens). We therefore doubt the presence of *I. obesa* comb. nov. in Nepal at present. Several specimens from Nepal are currently listed as *I. obesa* comb. nov. in OSF [7], but their true identity requires further investigation. It is likely that this taxon may comprise multiple distinct species.
***Ingrischana parlungana* Subedi et Skejo nom. nov.**
  **LSID.** urn:lsid:zoobank.org:act:0BD0CADF-CBE8-4B21-B903-F5CFB745FFA5
  =*Ingrischana serrifemoralis* (Zheng et Shi, 2009a) [20] comb. nov. (HT ♂ from China: Tibet: Bomi, deposited in SNNU); homonym of *Ingrischana serrifemoralis* (Zheng, 1998) comb. nov.
  =*Bannatettix serrifemoralis* Zheng et Shi, 2009a [20]
  *=Ergatettix serrifemoroides* Zheng et Shi, 2009b [21] syn. nov. (HT ♀ from China: Tibet: Nyingchi, deposited in SNNU); if not synonym of *I. parlungana* nom. nov., it is homonym with *I. serrifemoroides* (Zheng et Jiang, 2002) comb. nov. [15]
  **Distribution**: Known from a single locality in the basin of the Parlung River in Tibet (China) [20,21].
  **Notes**: *Ingrischana parlungana* nom. nov. is a new name proposed for *Bannatettix serrifemoralis* Zheng et Shi, 2009a from Tibet (China) because of secondary homonymy with *Ingrischana serrifemoralis* (Zheng, 1998) comb. nov. after the new combination is introduced. The adjective *parlunganus*, *parlungana*, *parlunganum* is based on the Latinized name of Parlung River, in whose basin the species occurs.
***Ingrischana serrifemora* (Deng, Zheng et Wei, 2008) comb. nov.**
  *=Ergatettix serrifemora* Deng, Zheng et Wei, 2008 [18]
  **Distribution**: Known only from the type locality in China, Guangxi [18] (Table 2).
  **Notes**: Transferred from the genus *Ergatettix* Kirby, 1914 [5].
***Ingrischana serrifemoralis* (Zheng, 1998) comb. nov.**
  =*Tetrix serrifemoralis* Zheng, 1998 [12]
  *=Tetrix serrifemora* [sic] Zheng, 1998, misspelling of *T. serrifemoralis*
  **Distribution**: China: reported from many localities in Yunnan (Hekou, Shizong, Yongren), and Guangxi (Bama, Dahua, Chongzuo, Shangsi) [58,65]
  **Notes**. Transferred from the genus *Tetrix* Latreille, 1802 [1].
***Ingrischana serrifemoroides* (Zheng et Jiang, 2002) comb. nov.**
  =*Tetrix serrifemoroides* Zheng et Jiang, 2002 [15]
  **Distribution**: China: known from two localities in Guangxi (Tiangyang, Longzhou) [15,58,65].
  **Notes**. Transferred from the genus *Tetrix* Latreille, 1802 [1].
***Ingrischana torulosinota* (Zheng, 1998) comb. nov.**
  =*Tetrix torulosinota* Zheng, 1998 [12]
  **Distribution**: China: reported from several localities in Yunnan (Mengla, Menglun, Yuangjiang) [65].
  **Notes**. Transferred from the genus *Tetrix* Latreille, 1802 [1].

### 3.2. Phylogeny

**Figure 11:**
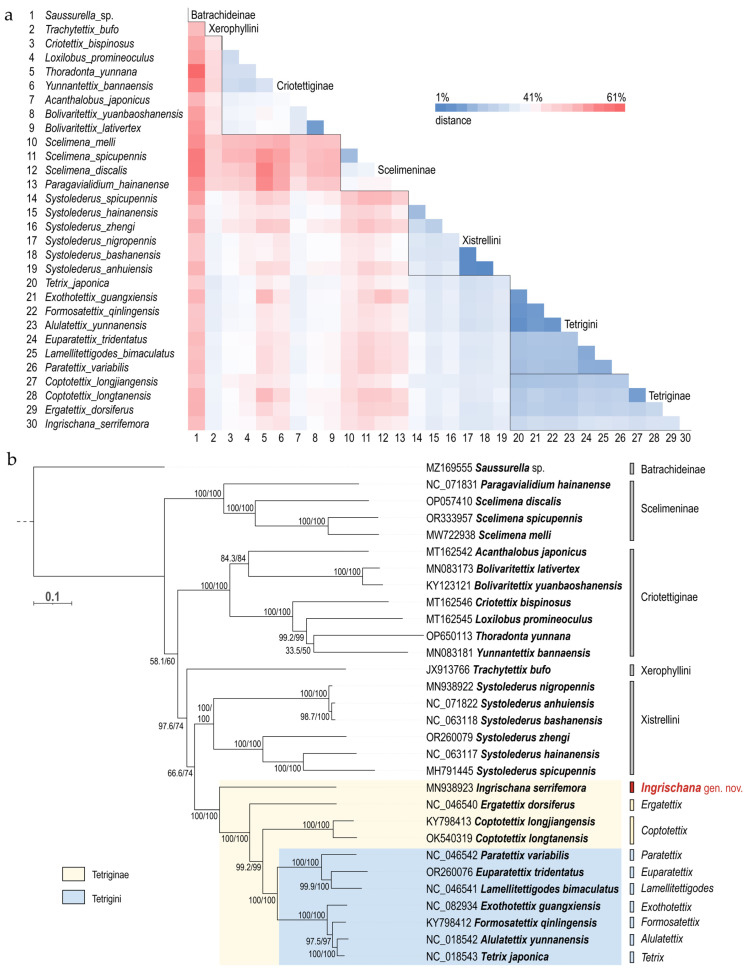
(**a**) Matrix of the distances between mitogenomes of the Tetrigidae species analyzed in this study; constructed in MEGA12 using the Maximum Composite Likelihood model. Framed are members of the same higher groups. (**b**) Maximum likelihood phylogram showing the relationship of 30 Tetrigidae species and the position of the genus *Ingrischana* **gen. nov**., reconstructed in IQ-TREE (GTR + G + I model, 1000 Bootstrap replicates). Node values show UFBoot and SH-aLRT, respectively.

We have observed lower pairwise distances between genera belonging to the same subfamily or tribe than between members of different subfamilies and tribes (Figure 11a). Subfamily Batrachideinae pairwise distance from other Tetrigidae was between 47% (against *Tetrix japonica*) and 61% (against *Thoradonta yunnana*). Subfamily Xerophyllini distance from other Tetrigidae was between 34% (against *Tetrix japonica*) and 47% (against *Scelimena melli*). Within Criotettiginae, we observed pairwise distances from 8% in the members of the same genus (e.g., two *Bolivaritettix* species) up to 41% between *Thoradonta* and *Bolivaritettix*; within Scelimeninae, we observed distances from 18% in two species of the same species group within the genus *Scelimena*, up to 41% between *Paragavialidium* and *Scelimena*; and within Xistrellini, we observed distances from 1% (e.g., closely related *Systollederus* species, or maybe even synonyms) up to 35%. Pairwise distance between genera traditionally assigned to the tribe Tetrigini—*Tetrix*, *Alulatettix*, *Formosatettix*, *Exothotettix*, *Paratettix*, *Euparatettix*, and *Lamellitettigodes*— ranged from 5% between *Alulatettix* and *Formosatettix* to 22% between *Euparatettix* and *Exothotettix*. Within the subfamily Tetriginae, distances span a broader range, from 5% as exemplified above, to 32% between *Ingrischana* gen. nov. and *Euparatettix*, *Paratettix*, and *Lamellitettigodes*, respectively (Figure 11a).

Distances between *Ingrischana serrifemora* comb. nov. and other Tetrigidae taxa are consistently high (28–49%). The lowest distances are observed in comparison with members of Tetriginae. Specifically, *I. serrifemora* comb. nov. shows the smallest divergence from *Tetrix japonica* (28%), followed by species of *Alulatettix* and *Formosatettix* (29%), *Exothotettix* and one species of *Coptotettix* (30%), and *Ergatettix dorsifera* (31%), the type species of *Ergatettix*. The highest divergence within Tetriginae reaches 32% between *Ingrischana* **gen. nov**. and representatives of four other Tetrigine genera (Figure 11a).

The phylogram with the best score (log likelihood: −223,207.951) inferred using the Maximum Likelihood method and GTR + G + I model of nucleotide substitutions, rooted on Batrachideinae (*Saussurella* sp.), is shown in Figure 11b. All the subfamilies and tribes—Scelimeninae (Scelimenini and Discotettigini), Criotettiginae, Xistrellini, Tetriginae, and Tetrigini—have been reconstructed as holophyletic, with strong Bootstrap values (100). Scelimeninae are reconstructed as a sister group to a clade containing Criotettiginae, Xerophyllini, Xistrellini, and Tetriginae, but with weak support. Criotettiginae are reconstructed as sister to a clade including Xerophyllini, Xistrellini, and Tetriginae, but also with weak support. Position of Xerophyllini as sister to Tetriginae and Xistrellini clade is moderately supported (Figure 11b).

*Ingrischana serrifemora* comb. nov. is always (Bootstrap 100/100) in the same clade with Tetriginae genera, but it never formed a cluster with any of the Tetriginae species. Importantly, *Ingrischana serrifemora* comb. nov. has never clustered with *Ergatettix dorsiferus*, the type species of *Ergatettix*. All the trees (Bootstrap 100/100) reconstructed *Ingrischana* gen. nov. as a basal genus within Tetriginae, sister to all other Eurasian genera. Within Tetriginae, all the Bootstrap values are constantly high (97–100). Genera *Tetrix*, *Alulatettix*, *Formosatettix*, and *Exothotettix* form one clade within Tetrigini; while *Paratettix*, *Euparatettix* and *Lamellitettigodes* form another. Two *Coptotettix* species were reconstructed as sister to Tetrigini; *Ergatettix* as sister to *Coptotettix* and Tetrigini ancestor; and *Ingrischana* gen. nov., as already stated, sister to all of them (Figure 11b).

## 4. Discussion

Herein, we establish *Ingrischana* gen. nov., a distinct lineage of Asian Tetriginae whose species were previously scattered among the genera *Bannatettix*, *Formosatettixoides*, *Ergatettix*, *Euparatettix*, *Paratettix*, and *Tetrix*. With 15 included species, the new genus is already among the more speciose genera of Tetriginae. The discovery of two additional species from Nepal further indicates that the diversity of this lineage remains underestimated and that additional undescribed taxa are likely to be found, particularly in underexplored regions of the Himalayan foothills and Indo-Burma biodiversity hotspot.

The taxonomy of Tetriginae remains challenging, and progress toward a natural classification has been gradual. Recent revisions, such as the work on African taxa related to *Paratettix* [66], demonstrate that careful reassessment of morphological characters can lead to the recognition of previously overlooked lineages. However, a comparable revision of Asian Tetriginae associated with *Paratettix* and *Tetrix* is still lacking. In this context, the present study represents an initial step toward resolving the taxonomy of large and heterogeneous Asian Tetriginae.

One of the central issues concerns the genus *Tetrix*, currently the most species-rich genus of Tetrigidae [7] and arguably one of the most taxonomically complex [67,68]. Molecular and morphological studies have demonstrated that *Paratettix* and *Tetrix* in their current circumscriptions are probably not monophyletic (e.g., [69,70]) and should be carefully reviewed. In light of these findings, we propose that species characterized by widened, densely setose fore and mid femora combined with serrated margins of the hind femora do not belong to *Tetrix*, but instead form a coherent and diagnosable lineage here recognized as *Ingrischana* gen. nov.

**Figure 12:**
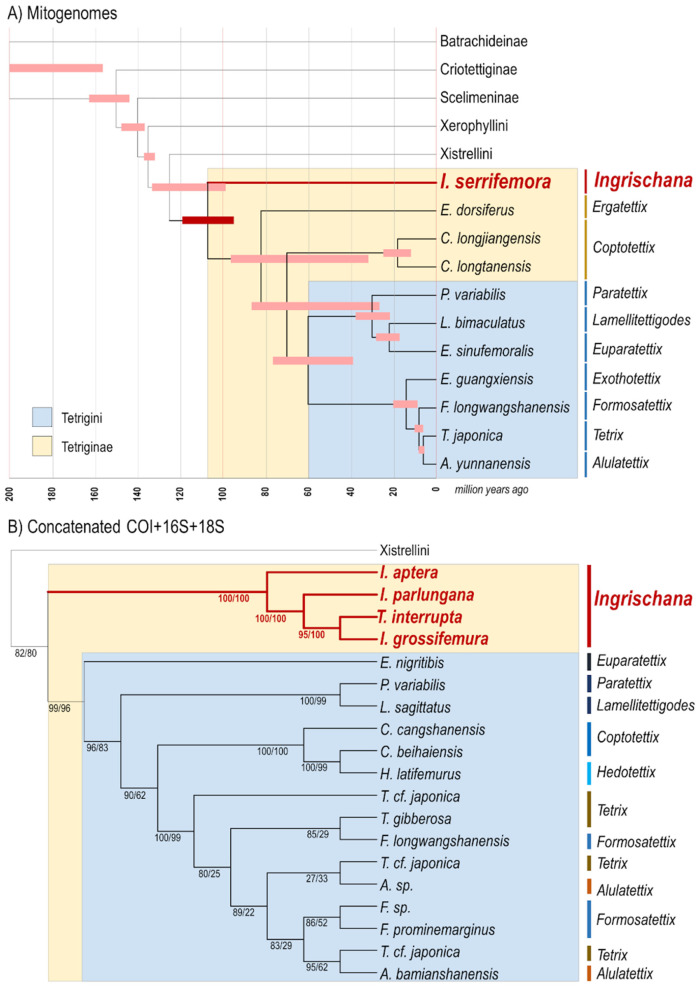
Further phylogenetic evidence on the distinctiveness of the genus *Ingrischana* gen. nov. from hitherto published studies. (**A**) A chronogram showing the position of *Ingrischana* gen. nov. on the Tetrigidae tree of life, as deduced on the basis of mitogenome phylogeny (15,086–17,643 bp length of sequences) [43] by the Bayesian Evolutionary Analysis Sampling Trees (BEAST). The potential span of each node is presented with a red bar, also following [43]. The pink bars on the chronogram represent uncertainty intervals, i.e., the time period during which the ancestor may have lived. (**B**) A cladogram showing the position of *Ingrischana* gen. nov., as deduced from the maximum likelihood analysis of COI + 16S + 18S concatenated sequence (3841 bp), using the GTR + I + G model with 1000 bootstrap replicates [70]. The numbers on the nodes represent Bootstrap values [70]. Note that there are many issues with Tetriginae classification and generic assignment.

The establishment of this new genus is supported not only by morphological apomorphies but also by phylogenetic evidence. Notably, *Ingrischana serrifemora* comb. nov. (originally described as *Ergatettix serrifemora*) is the only species of the genus for which a complete mitogenome has been sequenced (Li et al., 2020). In multiple independent phylogenetic reconstructions, including this study (Figure 11 and Figure 12), this species was recovered as a lineage distinct from both *Ergatettix dorsiferus* (Walker, 1871) [71], the type species of *Ergatettix*, and from representatives of *Tetrix* [40,42,43,72,73,74,75]. In several analyses, it appears as a sister to other Tetriginae genera, i.e., basal within Tetriginae, further emphasizing its distinctiveness. Divergence time estimates suggest that the lineage leading to *Ingrischana* gen. nov. may trace back to the Cretaceous (ca. 110–85 Ma [43], indicating a deep evolutionary history within Tetrigidae. It is worthy to mention that some more species have their COI, 16S, and 18S rRNA sequenced. These are *I. aptera* comb. nov., *I. parlungana* comb. nov., and *I. grossifemura* comb. nov. and they also formed a cluster sister to the rest of Tetriginae [70]. One more species fits in this cluster [70], that being *Tetrix interrupta*. However, Huang and colleagues [70] did not provide authorship of the species they studied, and there are three species named ‘*Tetrix interrupta*’ from China: the original *T. interrupta* Zheng, 2004 [76]; the unresolved junior homonym *T. interrupta* Deng, Zheng et Wei, 2009 [77]; and *T. interrupta* Zheng et Xu, 2010 [78] (resolved as *T. fuliginosoides* Deng, 2016 [58]). So far, we have not determined which ‘*Tetrix interrupta*’ belongs to *Ingrischana* **gen. nov**. Although broader molecular sampling of Asian Tetriginae is still required, current evidence is congruent with the morphological delimitation of *Ingrischana* gen. nov. as a separate genus. Interestingly, during the review of this paper, the transcriptome of *I. serrifemora* comb. nov. was published [79], and compared to several other Tetrigidae species. Observed expansion of metabolic and stress-response genes is hypothesized to represent an adaptation to exposed habitats on gravel, inhabited by this species, which likely exhibit large thermal and physiological stress [79].

Biogeographically, *Ingrischana* gen. nov. is distributed across the Oriental and Sino-Japanese realms [80]. The majority of species are known from southwestern China (Guangxi and Yunnan), with additional taxa occurring in Tibet, Myanmar, Thailand, and Nepal. The two new Nepali species extend the known range westwards along the southern Himalayan arc. *Ingrischana motbotawa* gen. et sp. nov. currently represents the westernmost confirmed occurrence of the genus. The nearest congeners are located more than 1200 km to the northeast (*I. parlungana* nom. nov.) and more than 1600 km to the southeast in Guangxi and Yunnan. This distribution pattern suggests either a historically wider and now fragmented range or insufficient sampling in intervening regions, particularly in northern India and adjacent Himalayan areas. Most Chinese species appear to have restricted and localized distributions [58], often occurring in close geographic proximity, which may reflect both high microendemism and complex topography in southwestern China.

A single record of *I. obesa* comb. nov. from Tanzania is currently listed in the Orthoptera Species File (specimen ID 1586187; GUID 7ea732d8-befd-48b9-9291-df12a23fc53a; Cigliano et al. 2026 [7]). This record is biogeographically implausible given the otherwise exclusively Asian distribution of the genus and likely represents either a misidentification or a labeling error. Clarification of this record will require re-examination of the material.

The species *Bannatettix barbifemura* Deng, Zheng et Wei, 2012 [81], exhibits significant morphological affinities with *Ingrischana* gen. nov., specifically in body size, vertex position, subparallel prozonal carinae, a tuberculated pronotum, serrated hind femora, and hairy mid-femora. However, *B. barbifemura* differs by having lower-inserted antennae, more elongated eyes, a more exserted head, and a more depressed prozona. While the leg morphology is strikingly similar, incorporating this species into *Ingrischana* gen. nov. risks creating a taxonomically heterogeneous genus. It is more probable that *B. barbifemura* represents a new genus closely related to *Ingrischana* gen. nov. Future research is required to determine whether these similarities reflect shared ancestry or are the result of convergent evolution.

The discovery of *I. aspinosa* gen. et sp. nov. is particularly noteworthy, as it represents only the second known tetrigid species with a reduced or “toothless” ovipositor, the other being *Edentatettix leyeensis* Deng, 2025 from Guangxi [73]. In the vast majority of Tetrigidae, ovipositor valves bear distinct serrations used for penetrating soil, moss, or plant tissues during oviposition [82]. The independent loss of serrations in two unrelated lineages likely represents convergent evolution. We hypothesize that the reduction observed in *I. aspinosa* gen. et sp. nov. may be associated with oviposition in consistently moist, soft substrates such as decomposing leaf litter or saturated soil in the humid lowlands of the Terai. This interpretation remains speculative but provides a testable ecological hypothesis.

Pronotal length variation (brachy-, pauro-, and macropronotal states) within *Ingrischana* gen. nov. also highlights the need for caution in tetrigid taxonomy. In several Asian Tetriginae, pronotal state has historically contributed to taxonomic confusion, with different morphs described as separate species (e.g., [68]). Our findings reinforce the view that pronotal length alone should not be treated as a primary generic or specific character without consideration of additional morphological and phylogenetic evidence.

Two questions arise that fall outside the scope of the present study but warrant future research. *What is the functional significance of the dense setosity on the fore and mid femora*? *What adaptive advantage might the serrated margins of the hind femora confer*? These traits may be associated with sensory functions, potentially enhancing mechanoreception, or they may play a role in intraspecific communication. Alternatively, they could contribute to camouflage or represent adaptations to other ecological specializations. By defining *Ingrischana* gen. nov. as a morphologically and phylogenetically distinct lineage, we provide a framework within which such evolutionary and functional questions can now be meaningfully addressed.

## 5. Conclusions

In conclusion, the recognition of *Ingrischana* gen. nov. contributes to the ongoing effort to achieve a more natural classification of Asian Tetriginae. Integrative approaches combining detailed morphology, expanded molecular sampling, and ecological data will be essential for resolving generic boundaries within Tetriginae and for understanding the evolutionary history of this diverse and still insufficiently explored group of pygmy grasshoppers.

## Figures and Tables

**Figure 3:**
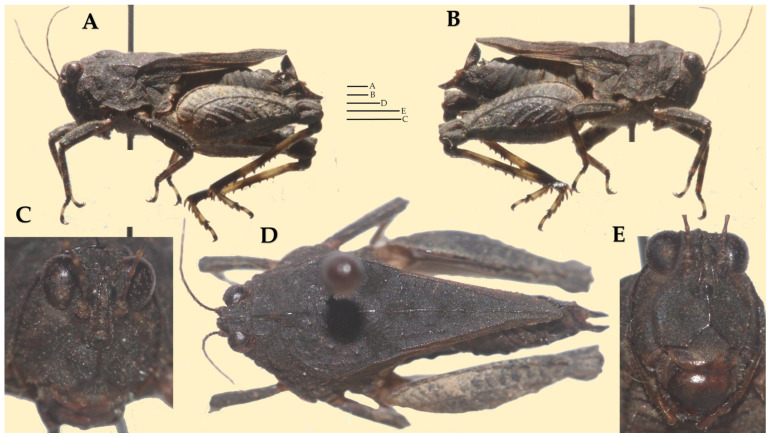
*Ingrischana aspinosa* gen. et sp. nov. female holotype. (**A**,**B**) habitus in lateral view; (**C**,**E**) head in frontal view; (**D**) habitus in dorsal view. Scale bar = 1 mm.

**Figure 4:**
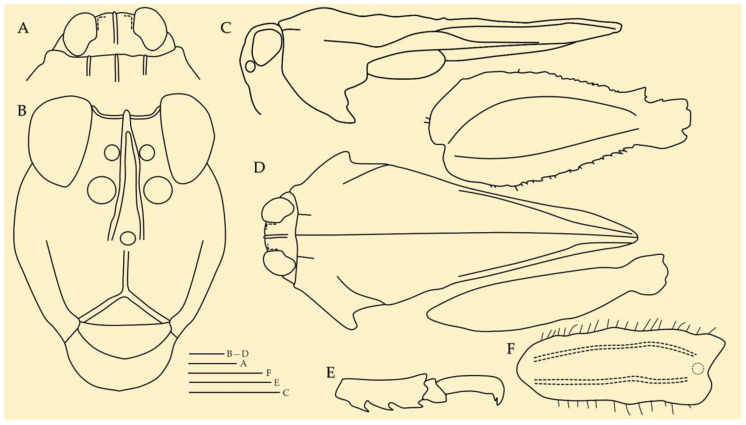
*Ingrischana aspinosa* gen. et sp. nov. female holotype. (**A**) head in dorsal view; (**B**) head in frontal view; (**C**) habitus in lateral view; (**D**) habitus in dorsal view; (**E**) hind tarsus in lateral view; (**F**) mid femur in lateral view. Scale bar = 1 mm.

**Figure 5:**
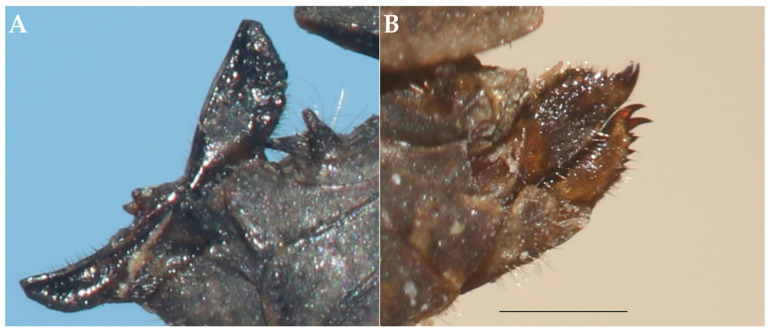
Ovipositor valves in lateral view. (**A**) *I. aspinosa* sp. nov. (**B**) *I. motbotawa* sp. nov. Scale bar = 1 mm.

**Figure 6:**
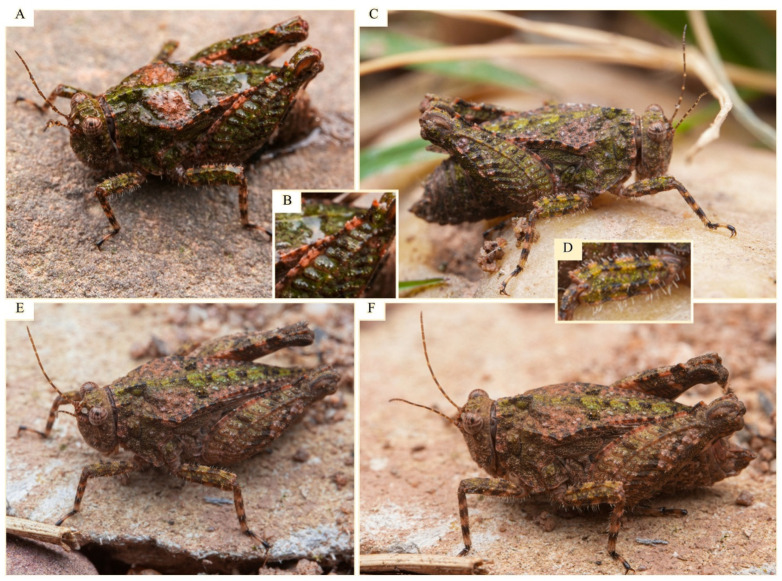
*Ingrischana barbifemura* comb. nov. in its natural habitat in Mengla County, Xishuangbanna Dai Autonomous Prefecture, Yunnan, China. (**A**) female in dorsolateral view, (**B**) hind femur serrations, (**C**) female in lateral view, (**D**) hairy mid femur, (**E**) individual (sex undeterminable from the photo) in dorsolateral view, (**F**) female in lateral view. Cropped photographs made by inaturalist user @simbason, observation IDs 241250129, 241250118, and 241250117 (CC-BY-NC licence).

**Figure 7:**
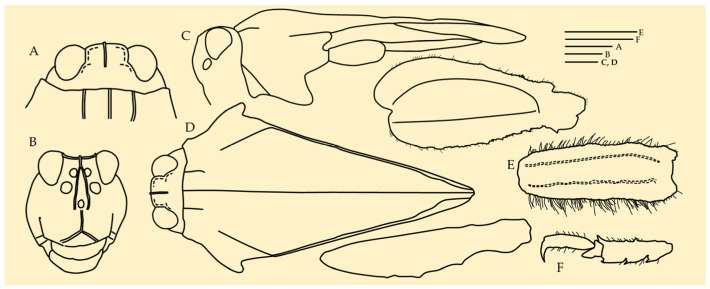
*Ingrischana motbotawa* gen. et sp. nov. female holotype. (**A**) head in dorsal view; (**B**) head in frontal view; (**C**) habitus in lateral view; (**D**) habitus in dorsal view; (**E**) mid femur in lateral view; (**F**) hind tarsus in lateral view. Scale bar = 1 mm.

**Figure 8:**
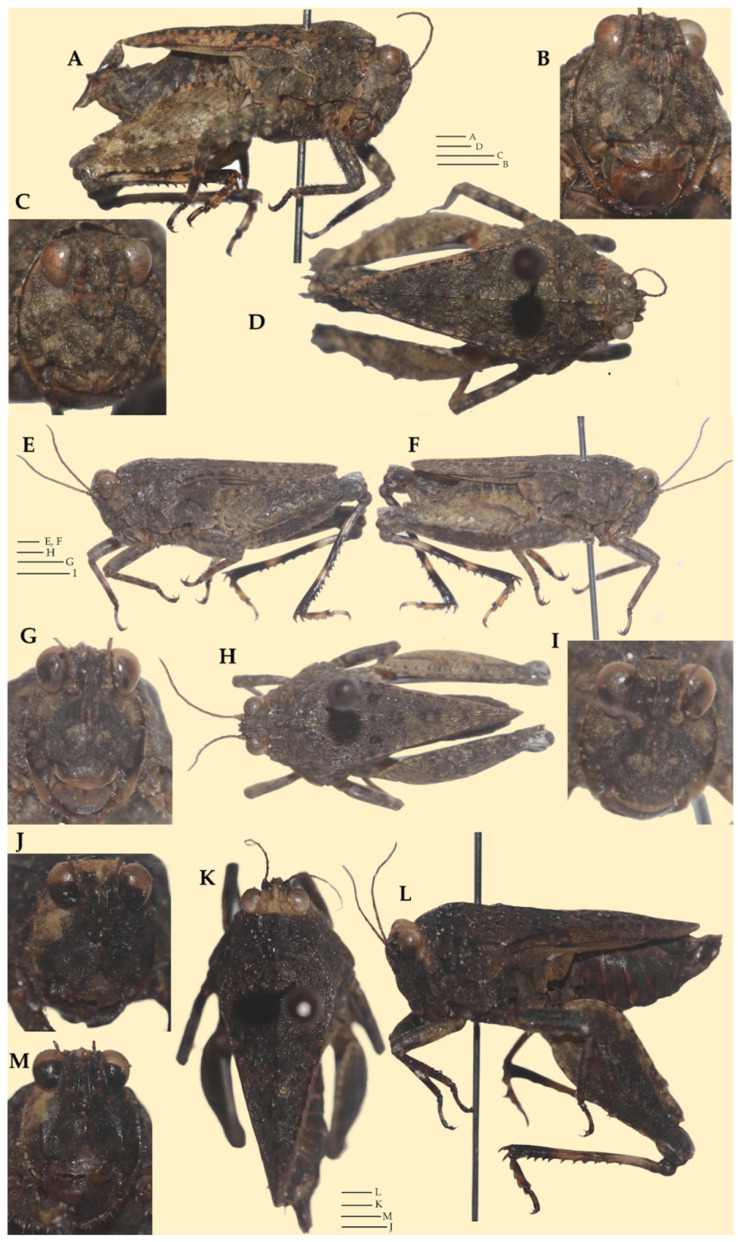
*Ingrischana motbotawa* gen. et sp. nov. Female holotype. (**A**) habitus in lateral view; (**B**,**C**) head in frontal view; (**D**) habitus in dorsal view. Scale bar = 1 mm. Male paratype. (**E**,**F**) habitus in lateral view; (**G**,**I**) head in frontal view; (**H**) habitus in dorsal view. Scale bar = 1 mm. Female paratype. (**J**,**M**) head in frontal view; (**K**) habitus in dorsal view; (**L**) habitus in lateral view. Scale bar = 1 mm.

**Figure 9:**
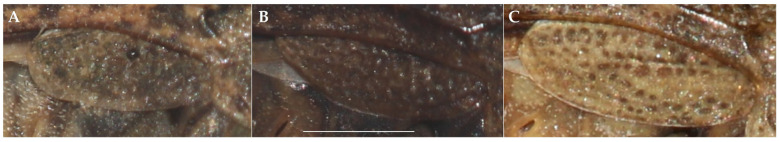
Variation in tegmina shape in *I. motbotawa* gen. et sp. nov. (**A**) Holotype (♀), (**B**) Paratype (♀), (**C**) Paratype (♂). Scale bar = 1 mm.

**Figure 10:**
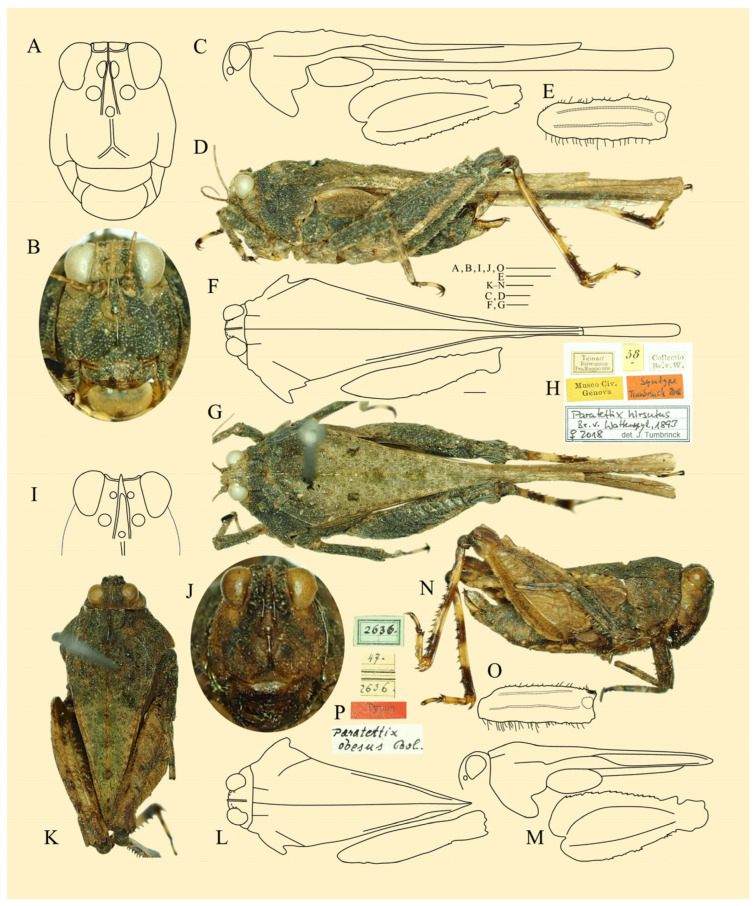
***Ingrischana obesa* (Bolívar, 1887) comb. nov.** (**A**–**H**) female syntype of *Paratettix hirsutus* Brunner von Wattenwyl, 1893 from NMW and (**I**–**P**) holotype female of *Paratettix obesus* Bolívar, 1887 from NMW. (**A**,**B**,**I**,**J**) head in frontal view; (**C**,**D**,**M**,**N**) habitus in lateral view; (**E**,**O**) mid femur; (**F**,**G**,**K**,**L**) habitus in dorsal view; (**H**,**P**) labels. Photo Josef Tumbrinck, drawings this study.

**Table 1:** List of species whose mitogenomes we studied in order to determine the position of *Ingrischana* gen. nov. on the Tetrigidae and Tetriginae tree of life. Included are data on higher classification (subfamily and/or tribe), as well as on GeneBank ID numbers.

Species	Classification	NCBI Accession	Length
*Saussurella* sp.	Batrachideinae	MZ169555	16,006 bp
*Criotettix bispinosus*	Criotettiginae	MT162546	14,838 bp
*Acanthalobus japonicus*		MT162542	14,247 bp
*Loxilobus promineoculus*		MT162545	15,025 bp
*Thoradonta yunnana*		OP650113	17,859 bp
*Bolivaritettix yuanbaoshanensis*		KY123121	14,905 bp
*Bolivaritettix lativertex*		MN083173	15,054 bp
*Yunnantettix bannaensis*		MN083181	15,167 bp
*Scelimena melli*	Scelimeninae	MW722938	14,598 bp
*Scelimena discalis*		OP057410	17,552 bp
*Scelimena spicupennis*		OR333957	16,069 bp
*Paragavialidium hainanense*		NC_071831	17,849 bp
*Trachytettix bufo*	Xerophyllini	JX913766	14,578 bp
*Systolederus spicupennis*	No subfamily assignment: Xistrellini	MH791445	15,262 bp
*Systolederus nigropennis*		MN938922	14,652 bp
*Systolederus hainanensis*		NC_063117	14,946 bp
*Systolederus bashanensis*		NC_063118	14,775 bp
*Systolederus anhuiensis*		NC_071822	15,458 bp
*Systolederus zhengi*		OR260079	15,398 bp
*Ingrischana serrifemora* comb. nov.	Tetriginae: no tribal assignment	MN938923	14,947 bp
*Ergatettix dorsiferus*		NC_046540	15,326 bp
*Coptotettix longtanensis*		OK540319	16,861 bp
*Coptotettix longjiangensis*		KY798413	14,495 bp
*Paratettix variabilis*	Tetriginae: Tetrigini	NC_046542	15,194 bp
*Lamellitettigodes bimaculatus*		NC_046541	15,221 bp
*Euparatettix tridentatus*		OR260076	15,086 bp
*Exothotettix guangxiensis*		NC_082934	17,643 bp
*Formosatettix qinlingensis*		KY798412	15,180 bp
*Tetrix japonica*		NC_018543	15,128 bp
*Alulatettix yunnanensis*		NC_018542	15,104 bp

**Table 2:** List of all *Ingrischana* **gen. nov**. species with type locality and information on the type material. HT—holotype.

Species	Type Locality	Name Bearing Type
*I. aptera* (Zheng et Ou, 2009) [19] comb. nov.	China: Yunnan: Ruili	HT ♂ (SNNU)
*I. aspinosa* gen. et sp. nov.	NEPAL: Bara: Tamagadhi	HT ♀ (ICAG)
*I. barbifemura* (Zheng, 1998) [12] comb. nov.	China: Yunnan: Menglun	HT ♂ (SNNU)
*I. curvimargina* (Zheng et Deng, 2004) [17] comb. nov.	China: Guangxi: Yizhou	HT ♀ (SNNU)
*I. dentifemura* (Zheng, Shi et Luo, 2003) [16] comb. nov.	China: Guangxi: Xincheng	HT ♂ (SNNU)
*I. grossifemura* (Zheng et Jiang, 1997) [11] comb. nov.	China: Guangxi: Nanning	HT ♂ (SNNU)
*I. jhapana* (Ingrisch, 2001a) [14] stat. rev.	NEPAL: Jhapa: Kakarbhitta	HT ♀ (SMF)
*I. longzhouensis* (Zheng et Jiang, 2000) [13] comb. nov.	China: Guangxi: Longzhou	HT ♀ (SNNU)
*I. motbotawa* gen. et sp. nov.	NEPAL: Kapilvastu: Lake Brija	HT ♀ (ICAG)
*I. obesa* (Bolívar, 1887) [3] comb. nov.	MYANMAR (ambiguous, see in text)	HT ♀ (NMW)
*I. parlungana* nom. nov.	China: Tibet: Bomi	HT ♀ (SNNU)
*I. serrifemora* (Deng, Zheng et Wei, 2008) [18] comb. nov.	China: Guangxi: Luocheng	HT ♀ (SNNU)
*I. serrifemoralis* (Zheng, 1998) [12] comb. nov.	China: Yunnan: Yongren	HT ♀ (SNNU))
*I. serrifemoroides* (Zheng et Jiang, 2002) [15] comb. nov.	China: Guangxi: Longzhou, Nonggang	HT ♀ (SNNU)
*I. torulosinota* (Zheng, 1998) [12] comb. nov.	China: Yunnan: Jinping, Mengla	HT ♀ (SNNU)

**Table 3:** Comparative morphological table (first part) comparing thirteen traits in all 15 species assigned to *Ingrischana* gen. nov. Measurement data were taken from [3,11,12,13,14,15,16,17,18,19,20,21].

Species	Vertex/Eye Ratio (Sex)	Anterior Margin of Vertex in Comparison to Eyes	Anterior Margin of Pronotum	Prozonal Carinae (Anterior to Posterior)	Median Carina of Pronotum (Lateral View)	Pronotum Surface	Pronotum Length
*I. aptera* comb. nov.	1.1 (♂)	in level	straight	subparallel	arched, then flat	finely granulated	brachy
*I. aspinosa* sp. nov.	1.25 (♀)	not reaching	slightly produced	subparallel	arched, then slightly undulated	finely granulated	brachy
*I. barbifemura* comb. nov.	1.3 (♂)	in level	straight	subparallel	arched, then undulated	tuberculated	brachy
*I. curvimargina* comb. nov.	1.5 (♀)	in level	straight	weakly converging	arched, then undulated	smooth	brachy
*I. dentifemura* comb. nov.	0.8 (♂)	in level	straight	subparallel	arched, then flat	smooth	pauro
*I. grossifemura* comb. nov.	1.25 (♂)	not reaching	straight	subparallel	arched, then strongly undulated	finely nodulated	brachy
*I. jhapana* stat. rev.	1.2 (♀)	not reaching	straight	subparallel	arched, then flat	densely granular	brachy
*I. longzhouensis* comb. nov.	≈1 (♀)	not reaching	straight	subparallel	arched, then flat	finely nodulated	brachy
*I. motbotawa* sp. nov.	1.4 (♀), 1.3 (♂)	in level	slightly produced	parallel to slightly converging	arched, then flat	finely granulated/wrinkled	brachy
*I. obesa* comb. nov.	1.4 (♀)	not reaching	straight	weakly converging	slightly arched, then flat	tuberculated	brachy to pauro
*I. parlungana* nom. nov.	0.9 (♀)	not reaching	straight	subparallel	slightly arched/undulated, then flat	finely tuberculated	brachy
*I. serrifemora* comb. nov.	0.9 (♀)	not reaching	straight	weakly converging	slightly arched then undulated	finely tuberculated	brachy
*I. serrifemoralis* comb. nov.	≈1 (♀)	not reaching	straight	subparallel	undulated, then flat	finely tuberculated	pauro
*I. serrifemoroides* comb. nov.	≈1 (♀)	in level	straight	weakly converging	weakly undulated, then flat	finely tuberculated	pauro
*I. torulosinota* comb. nov.	1.3 (♀)	in level	straight	subparallel	arched then flat	densely tuberculated	pauro

**Table 4:** Comparative morphological table (second part) comparing thirteen traits in all 15 species assigned to *Ingrischana* gen. nov. Measurement data were taken from [3,11,12,13,14,15,16,17,18,19,20,21]. Abbreviation “n/v” means the character is not easily visible because of the preparation.

Species	Hind Wing Length	Mid Femora Length/Width Ratio (Sex)	Hind Femora Length/Width Ratio (Sex)	Serrations on Hind Femora (Dorsal/Ventral)	Ovipositor Spines	Size (mm)
*I. aptera* comb. nov.	absent	2.8 (♂)	2.3–2.5 (♂)	large/absent	fine	8.0–8.3 (♀), 7.0–7.5 (♂)
*I. aspinosa* sp. nov.	slightly abbreviated	3.0 (♀)	2.4 (♀)	fine/fine	absent	13.45 (♀)
*I. barbifemura* comb. nov.	slightly abbreviated	2.2 (♂)	2.2 (♀,♂)	fine/fine	fine	8.5–11 (♀), 8.0–8.5 (♂)
*I. curvimargina* comb. nov.	slightly abbreviated	3.9 (♀)	3.2 (♀)	large/large	fine	10.5 (♀)
*I. dentifemura* comb. nov.	developed	3.0 (♂)	2.9 (♂)	large/fine	N/A (male)	9 (♂)
*I. grossifemura* comb. nov.	slightly abbreviated	2.9 (♂)	2.2(♂)	large/absent	N/A (male)	9 (♂)
*I. jhapana* stat. rev.	slightly abbreviated	3.3 (♀)	2.3 (♀)	fine spines alternating with large	n/v	8.52 (♀)
*I. longzhouensis* comb. nov.	slightly abbreviated	3.5 (♀)	3.7 (♀)	fine/fine	fine	11–12 (♀), 10 (♂)
*I. motbotawa* sp. nov.	slightly abbreviated	3 (♀), 2.3 (♂)	2.7 (♀), 2.4 (♂)	fine/large	fine	11.9–12.16 (♀), 10.55 (♂)
*I. obesa* comb. nov.	slightly abbreviated	3.7 (♀)	2.4 (♀)	large/fine	fine	9.34 (♀)
*I. parlungana* nom. nov.	developed	2.7 (♀)	2.6 (♀)	large/large	fine	8.0–11.0 (♀), 7.5–8.5 (♂)
*I. serrifemora* comb. nov.	developed	3.0 (♀)	2.5 (♀)	large/fine	fine	8.0–8.5 (♀), 6.5–6.7 (♂)
*I. serrifemoralis* comb. nov.	developed	3.3 (♀)	2.6 (♀)	large/large	fine	12.5 (♀), 11 (♂)
*I. serrifemoroides* comb. nov.	developed	2.7 (♀)	3.0 (♀)	fine/fine	fine	11.5 (♀), 10 (♂)
*I. torulosinota* comb. nov.	developed	2.5 (♀)	2.6 (♀)	fine/fine	fine	10 (♀)

**Table 5:** Measurements (in mm) of *I. motbotawa* gen. et sp. nov., *I. aspinosa* gen. et sp. nov., and *I. obesa* comb. nov. Abbreviation “n/v” means the character is not easily visible because of the preparation; “HT” means holotype, “PT” means paratype.

Body Parts	*I. motbotawa* gen. et sp. nov.			*I. aspinosa* gen. et sp. nov.	*I. obesa* comb. nov.	
	HT (♀)	PT (♂)	PT (♀)	HT (♀)	HT (♀)	Jhuwani (♀)
Body length	12.16	10.55	11.90	13.45	9.34	7.76
Vertex width	0.89	0.88	0.96	0.77	0.78	0.59
Eye width	0.61	0.69	0.69	0.63	0.56	0.51
Scutellum width	0.32	0.32	0.26	0.23	0.28	0.23
Pronotum length	8.97	9.44	10.16	10.15	8.14	8.21
Pronotum lobe width	5.16	4.63	5.10	4.87	4.27	3.40
Pronotum height	3.27	3.46	3.89	3.02	2.74	2.34
Tegmen length	1.84	1.85	2.02	2.35	n/v	1.69
Tegmen width	0.79	0.72	0.60	0.76	0.81	0.75
Alae length	4.89	6.01	5.33	6.25	5.06	9.95
Fore femur length	2.39	2.53	2.43	2.56	1.73	1.52
Fore femur width	0.67	0.69	0.68	0.78	0.57	0.44
Mid femur length	2.71	2.36	2.87	2.98	2.45	1.90
Mid femur width	0.95	1.03	0.91	0.98	0.66	0.56
Hind femur length	5.98	6.91	7.35	7.27	5.70	4.73
Hind femur width	2.34	2.83	2.63	3.02	2.41	1.99
Hind tibia length	5.13	6.42	6.23	6.30	5.06	4.12
Hind basal tarsal segment length	1.20	1.08	1.21	1.23	1.10	0.80
Hind apical tarsal segment length	0.77	0.90	0.83	0.77	0.74	0.61
Subgenital plate length	n/v	0.85	n/v	n/v	n/v	n/v
Subgenital plate width	n/v	0.51	n/v	n/v	n/v	n/v
Ovipositor dorsal valve length	1.31	N/A (male)	1.57	1.43	1.16	0.90
Ovipositor dorsal valve width	0.56	N/A (male)	0.56	0.61	0.48	0.37
Ovipositor ventral valve length	1.39	N/A (male)	1.49	1.67	1.02	0.66
Ovipositor ventral valve width	0.38	N/A (male)	0.39	0.35	0.30	0.27

## Data Availability

The datasets generated and/or analyzed during the current study are available from the corresponding author upon reasonable request.

## Abbreviations

Museum Abbreviations	
ANHM	Annapurna Natural History Museum, Pokhara, Kaski, Nepal
ICAG	Insect Collection of Agriculture Science Center, Ghyalchok, Gorkha, Nepal
NMW	Naturhistorisches Museum, Wien, Austria
SMF	Senckenberg Forschungsinstitut und Naturmuseum, Frankfurt, Germany
SNNU	Museum of Flora and Fauna of Shaanxi Normal University, Shaanxi, China
Other Abbreviations	
ca.	Circa
comb. nov.	New combination
gen. nov.	New genus
m a.s.l.	Meters above sea level
mm	Millimeters
nom. nov.	New name
sp. nov.	New species
stat. rev.	Reviewed status
